# European respiratory monographs: community-acquired pneumonia

**DOI:** 10.1186/cc13972

**Published:** 2014-07-04

**Authors:** Michael S Niederman

**Affiliations:** 1 Department of Medicine, Winthrop-University SUNY at Stony Brook, 222 Station Plaza North, Suite 509, Mineola, NY 11501, USA

## Abstract

**Book details:**

Chalmers JD, Pletz MW, Aliberti S (Eds): *Community-Acquired Pneumonia.* European Respiratory Monographs. Sheffield: European Respiratory Society; 2014, ISBN 978-1-849-84049-1.

## 

Community-acquired pneumonia (CAP) is the most common cause of death from infectious disease worldwide, and over the past 25 years there has been dramatic progress in research about all aspects of this disease. The European Respiratory Monograph devoted to this topic is a comprehensive review of the topic aimed at a broad range of health professionals, including critical care physicians.

The 20 chapters of this monograph span topics from CAP epidemiology to bacteriology, pathogenesis, risk assessment, diagnosis, therapy and prevention. As is emphasized in the introduction by Tobias Welte, the future management of this illness will focus on prevention and personalized care, modified to account for individual variations in the immune response. However, for the present we need to manage this disease on a daily basis, and the discussions in the book provide much practical information for those assessing and treating patients with severe CAP.

The topics of interest to ICU physicians include: assessment of illness severity, with a discussion of prognostic scoring systems; empiric therapy selection, with a good review of the role of dual-agent therapy for severe pneumonia; management of acute respiratory failure, with particular caution about the judicious use of non-invasive ventilation for pneumonia patients, and the need for timely intubation in selected patients; and the management of sepsis due to CAP. The discussion of sepsis nicely summarizes the literature about early goal-directed therapy, the selection of the type of fluid to support blood pressure, the use of vasopressors, and the role of corticosteroids in sepsis. Several other chapters include topics of vital interest in the ICU, such as the assessment of response to therapy, the use of non-antibiotic therapies, and the role of macrolides as anti-inflammatory agents. There is also an important discussion of the cardiovascular complications of CAP, a recently appreciated area of interest, which can explain some of the long-term sequelae of this illness.

Although *Community-Acquired Pneumonia* is comprehensive and inclusive, most of the authors are European and they have interpreted some of the data and controversies from a perspective that is not always consistent with a North American viewpoint. Most notable among these issues is the commentary about the lack of utility of the concept of healthcare-associated pneumonia, yet very few concrete recommendations are given about how to manage this population of patients, if they are to be considered suffering a form of CAP. Other omissions include a limited discussion of the epidemiology of pneumonia in the ICU, the role of dual antibiotic therapy in pneumococcal bacteremia, the management of influenza in the ICU, and the importance of hospital-based immunization.

On balance, *Community-Acquired Pneumonia* is an excellent, balanced and comprehensive summary of an important and common illness; providing a great resource for physicians of all types, including those who work in critical care.

## Abbreviations

CAP: community-acquired pneumonia.

## Competing interests

The author declares that he has no competing interests.

